# Human infestations with *Fasciola hepatica* in Austria between 1955 and 2025: data from a low-incidence country

**DOI:** 10.1051/parasite/2026047

**Published:** 2026-08-26

**Authors:** Peter Mikosch, Eva Trifina-Mikosch, Caroline Schwarz, Herbert Auer

**Affiliations:** 1 Medical University of Vienna, Teaching Unit Vienna Austria; 2 Department of Internal Medicine 2, General Hospital Mistelbach-Gänserndorf Liechtensteinstraße 67 2130 Mistelbach Austria; 3 Karl Landsteiner Institute of Inflammation and Metabolism Mistelbach Austria; 4 Klinik Ottakring, Department of Internal Medicine IV Vienna Austria; 5 Medical Parasitology, Institute of Specific Prophylaxis and Tropical Medicine, Center of Pathophysiology, Infectiology and Immunology and Tropical Medicine, Medical University of Vienna Vienna Austria

## Abstract

Although rare, human *Fasciola hepatica* (FH) infestations represent a clinically relevant parasitosis that can cause significant clinical manifestations and functional limitations, particularly affecting the liver. Laboratory tests typically reveal elevated white blood cell counts and absolute or relative eosinophilia. The Department of Medical Parasitology at the Institute for Specific Prophylaxis and Tropical Medicine, Center of Pathophysiology, Infectiology and Immunology, Medical University of Vienna, is the national reference center for FH infestations in Austria. In this study, we assessed all confirmed human FH infestation cases between 1955 and 2025 and retrospectively analyzed their demographic, clinical, laboratory, and imaging data. In all, 64 human FH infestations were recorded. Although some patients were asymptomatic, most had fever, abdominal pain, hepatosplenomegaly, and elevated liver parameters. Pancreatitis, diarrhea, pulmonary symptoms, weight loss, or skin lesions, mainly urticaria, were present in a few cases. During the observation period, the diagnosed cases gradually increased decade by decade, largely due to improved diagnostic tests for FH. However, during the last 10 years, a decline in new diagnosed cases has been observed. Until the 1980s, cases concerned only individuals born in Austria. Since the 1990s, there have been case reports concerning people with a migration background, reflecting demographic changes. The distribution of FH cases within Austria was found to be unequal, with certain regions reporting higher incidence (Salzburg, Upper Austria, and Vorarlberg). *Fasciola hepatica* infestation is a rare parasitosis in Austria. However, it should be considered as a differential diagnosis, especially among migrants and travelers returning from endemic regions presenting with eosinophilia and abdominal pain.

## Abstract (fr)

Bien que rares, les infestations humaines par *Fasciola hepatica* (FH) constituent une parasitose cliniquement significative, susceptible d'entraîner des troubles cliniques importants et des limitations fonctionnelles, affectant principalement le foie. Les analyses de laboratoire révèlent généralement une hyperleucocytose ainsi qu'une éosinophilie absolue ou relative. Le Département de parasitologie médicale de l'Institut de prophylaxie spécifique et de médecine tropicale (relevant du Centre de physiopathologie, d'infectiologie et d'immunologie de l'Université de médecine de Vienne) fait office de centre national de référence pour les infestations par FH en Autriche. Cette étude a recensé tous les cas confirmés d'infestation humaine par FH entre 1955 et 2025 et a analysé rétrospectivement les données démographiques, cliniques, biologiques et d'imagerie associées. Au total, 64 cas d'infestation humaine par FH ont été enregistrés. Si certains patients étaient asymptomatiques, la majorité présentait de la fièvre, des douleurs abdominales, une hépatosplénomégalie et une élévation des paramètres hépatiques. Des cas de pancréatite, de diarrhée, de symptômes pulmonaires, de perte de poids ou de lésions cutanées (principalement de l'urticaire) ont également été observés chez quelques patients. Au cours de la période d'étude, le nombre de cas diagnostiqués a progressivement augmenté d'une décennie à l'autre, en grande partie grâce à l'amélioration des tests diagnostiques pour la FH. Toutefois, une diminution du nombre de nouveaux cas diagnostiqués a été observée au cours des dix dernières années. Jusqu'aux années 1980, les cas concernaient uniquement des personnes nées en Autriche. Depuis les années 1990, des cas impliquant des personnes issues de l'immigration ont été signalés, reflétant ainsi les évolutions démographiques. La répartition géographique des cas de FH en Autriche était inégale, certaines régions (Salzbourg, Haute-Autriche et Vorarlberg) signalant une incidence plus élevée. L'infestation par *Fasciola hepatica* est une parasitose rare en Autriche. Elle doit néanmoins être envisagée dans le cadre d'un diagnostic différentiel, en particulier chez les migrants et les voyageurs de retour de zones d'endémie présentant une éosinophilie et des douleurs abdominales.

## Introduction

Fascioliasis is caused by two trematode parasites: *Fasciola hepatica* [FH] and *Fasciola gigantica*. FH originated in the Near-Middle East and has since spread worldwide [42, 44, 71], including throughout Europe. The German zoologist Peter Simon Pallas was the first to discover sheep liver flukes (*Fasciola hepatica*) in the hepatic ducts of a female patient during an autopsy in 1760 [26]. However, archaeological excavations have proved that FH has parasitized sheep, cattle and other herbivores, and humans since ancient times [12, 17].

Nowadays, FH is considered a relevant parasitosis worldwide [33, 59], with between 35 and 72 million people being infected and an estimated population of 180 million people at risk of infection, according to the World Health Organization (WHO) [20, 63, 74]. In ruminants, 350 million cattle and 250 million sheep are at risk of acquiring FH, reflecting a notable concern in the farming industry [48]. FH in animals and humans is usually seen in endemic areas, where the environmental conditions promote FH dissemination. The main endemic areas include Central and South America, Asia, the Middle East, Australia, China, and parts of Africa [16, 34, 42, 44, 45, 52]. In Europe and the Mediterranean region, mainly France [13, 60], Spain [23], Türkiye [32, 68], and Egypt are affected [16, 41, 53]. Although well-known to veterinarians and physicians across Europe and the Mediterranean region, human fascioliasis remains an underestimated zoonosis and a relevant health concern in many countries [16, 45, 52].

Fascioliasis is also caused by a second species, *Fasciola gigantica*. Different to the cosmopolitan geographic distribution of FH, *F. gigantica* is restricted to tropical and subtropical regions of Asia and Africa [42, 44]. In some of these regions, the presence of *F. gigantica* overlaps with that of FH, thus enabling infestations with both fluke types in these areas [29, 44].

FH has a complex life cycle. Adult flukes measure up to 35 mm long and 15 mm wide. They live in the bile ducts of ruminants, as well as other herbivorous and omnivorous animals (including humans), which function as final hosts for FH. The flukes shed up to 10,000 eggs daily into the host’s intestine, which are consecutively distributed through the host’s feces into the environment. In freshwater, miracidia emerge from the eggs and penetrate the body of the lymnaeid snail (*Galba truncatula* in Central Europe). After developing into sporocysts, rediae, and cercariae, the latter emerge from the snail and attach to plants. There, they lose their tails and encyst (metacercariae). Infestation of the final hosts can occur through the consumption of grass (animals) or raw aquatic vegetables, such as watercress, or bear leek, but can also be acquired through fruit lying in moist grass. Another extremely uncommon form to acquire FH is by drinking water contaminated by metacercariae.

In the final host, the metacercariae excyst in the duodenum and penetrate the intestinal wall. Then, they pass through the abdominal cavity, bore into the liver parenchyma, and eventually migrate through the liver into the biliary tract, where they mature into adults [34]. For a complete description of the life cycle of FH, please see: https://www.cdc.gov/dpdx/fascioliasis/index.html [19]. The lifecycle of *F. gigantica* is rather similar to that of FH, but the parasite uses other intermediate snail hosts and the times for development in the intermediate and final hosts are different.

Humans are fully compatible hosts for fascioliasis and can transmit the parasite, although they do not usually play a major role in transmission, particularly in developed countries. However, in conditions where open defecation is common (some rural areas in developing countries), human feces containing *Fasciola* eggs can contaminate the environment and thus occasionally contribute to the lifecycle of FH. The incubation period varies between 1 and 12 weeks. Fascioliasis is typically accompanied by eosinophilia and leukocytosis and often leads to cholestasis and/or elevated transaminases. Clinically, FH is characterized by abdominal pain, fever, malaise, and gastrointestinal symptoms like jaundice, recurrent biliary colic, and urticaria [1, 14, 15, 34, 65, 68]. One month after clinical onset, fascioliasis becomes chronic, with jaundice due to obstruction of the bile ducts by the parasites [42, 44, 62]. Severe gastroenterological disorders like cholangiocarcinoma or colon tumors can be induced by FH infestation [21, 56, 66], or the infestation can mimic these diseases [10, 35, 36, 40, 57, 73, 76, 77]. More rarely, neurological symptoms can occur either directly from flukes invading the central nervous system or indirectly from fluke secretions causing inflammation [3, 38, 62].

Diagnosis of human fascioliasis is based on clinical symptoms, elevated eosinophil counts, and parasitological examination, as well as patients’ origin, travel history, and potential risk behavior (i.e., raw vegetable consumption).

Over the years, FH diagnosis has evolved from simple microscopy to sophisticated molecular and serological assays [44, 43, 63, 74]. The first tests assessing FH infestation relied on visual stool examination and confirmation of the parasite’s presence by identifying eggs in feces. However, eggs only appear 8–12 weeks post-infection and egg shedding is intermittent, often requiring multiple samples for a confirmed diagnosis. False negative results may thus occur [63]. Today, parasitological stool analysis plays only a secondary role in the FH diagnostic work-up due to the availability of serological test [44].

Modern testing is based on serology, with immunodiagnostics offering early detection and high specificity [45]. Serological tests are the most useful tool during the acute phase, as antibodies can be detected 2–4 weeks after infection. FH screening is performed by ELISA, which is highly sensitive for detecting infection long before eggs are shed. Western blot assays (e.g., using recombinant FhSAP2 antigen) offers specificity of about 98% and can be used to confirm positive ELISA results. These assays show lower cross-reactivity than other serological methods. The Western blot technique has been used widely in recent years for fascioliasis diagnosis [51]. Immunoblot testing is recommended by the Haute Autorité de Santé in France and the US Centers for Disease Control for serodiagnostic confirmation [19, 28]. The detection of *Fasciola* DNA using various molecular methods does not only allow rapid and accurate diagnosis of fascioliasis, but also makes it possible to identify the specific species involved in the infestation [63]. These tests are therefore particularly relevant for areas where both FH and *F. gigantica* co-occur [29, 63]. The anthelminthic compound of choice for treating human FH infestation is triclabendazole, administered orally at 10 mg/kg/day b.i.d in equal doses [4, 34, 75].

FH is a common parasite among Austrian cattle [18, 39], particularly in the provinces of Vorarlberg, Tyrol [46], Salzburg, Upper Austria, and Styria [18, 39, 46, 58, 67]. In Tyrol, the overall herd prevalence of FH in cattle was reported to be 73% (range 17–97%), as assessed by testing bulk milk samples [46]. In another study from Carinthia, ELISA testing of milk samples showed 42.7% and 44.2% positive results, and testing of blood samples showed 43% and 45.2% positive for FH, based on two different assays applied, respectively, affecting more than 90% of the involved farms [18]. A study evaluating the endoparasitic fauna of sika deer in Austria found FH within the gastro-intestinal tract of 4.6% of animals [58]. Although humans are not the primary targets of FH infestations, they may also be affected, mainly in rural areas [60].

Humans are rarely affected by FH in Austria. Johann Gottfried Bremser, a helminthologist at the “K.u.K. Naturalienkabinett” in Vienna, stated in his monograph from the year 1819 that FH was an exceedingly rare parasite of humans in Austria at the time [11]. Since then, only a few case reports and series on FH occurrence in Austria have been reported [3–9, 24, 27, 37, 38, 49, 67, 70, 75].

This article aims to retrospectively analyze FH cases diagnosed by the Austrian reference center for FH over the last 70 years, and to shed further light on this rare parasitosis within Austria.

## Materials and methods

### Ethics statement

The project was performed according to the Declaration of Helsinki, Good Clinical Practice guidelines, and local requirements. The study protocol was approved by the institutional review board (Ethics committee of the Federal State of Lower Austria, GS3-EK-12/954-2025).

### Study design and aims

This study aimed to investigate the incidence of human FH infestation in Austria between 1955 and 2025 through a retrospective data analysis of all confirmed cases nationwide recorded by the Center of Pathophysiology, Infectiology and Immunology at the Medical University Vienna (MUW), which is the national reference center for FH infestations. All confirmed fascioliasis cases diagnosed in Austria were reported to this center, regardless of the original site reporting the diagnosis. Additionally, the study aimed to evaluate diagnostic findings (FH diagnosis method, laboratory parameters), factors predisposing to FH infestation (eating habits, travel activities), clinical symptoms, and demographic parameters (Austrian region, Austrian/non-Austrian origin, year/decade of diagnosis) in affected patients. Non-Austrian patients were, as far as data were available, allocated to the Austrian federal state in which they were living after their arrival.

### Case definitions and inclusion criteria

As this study covers a period of 70 years, sampling methods and materials changed multiple times throughout the observation period. Confirmed cases were defined according to the respective diagnostic standards at the time of diagnosis. The specific tests used are summarized in Table 1. In addition to serological testing, coprological examination (accumulation by Telemann) was performed to detect FH eggs (Table 1). All individuals with confirmed FH infestation during the observation period were included in the study, regardless of age, citizenship, descent, and institution of initial diagnosis in Austria. There were no exclusion criteria.

**Table 1:** Serological tests used for the diagnosis of human *Fasciola hepatica* infestation between 1955 and 2025 at the Institute of Parasitology and Microbiology at the Medical University Vienna, Vienna, Austria.


**1955–1989**
– IIFT (Indirect Immunofluorescence Test) – in-house test
+ antigen: *Fasciola hepatica* tissue section (own preparation)
+ conjugate: FITC-anti-human gammaglobulin (Behring); dilution medium: PBS (pH 7.2); dilution used: 1:40 (Auer *et al*., 1981)
and/or
– IHA (Indirect Hemagglutination Assay)
+ Test kit “Distomatose Fumouze” (Laboratoire Fumouze, France)
and/or
– CFR (Complement Fixation Reaction)
+ antigen: lyophilized liver fluke antigen (Institut Pasteur); dilution medium: DBP; dilution used: 1:100 (Auer *et al*., 1981)
**1990–2001**
– ELISA – in-house test
+ antigen: lyophilized liver fluke antigen (Institut Pasteur); dilution medium: carbonate buffer (pH 9.6); dilution used: 1:100;
+ conjugate: peroxidase-conjugated anti-human gammaglobulin (Cappel Laboratories); dilution medium: PBS (pH 7.2) + 0.05% Tween 20 + 2% bovine albumin; (Auer *et al*., 1981)
– CIEP (Counterimmunoelectrophoresis)
+ antigen: Institut Pasteur
**2002–2013**
– ELISA – in-house test
+ antigen: lyophilized liver fluke antigen (Institut Pasteur); dilution medium: carbonate buffer (pH 9.6); dilution used: 1:100;
+ conjugate: peroxidase-conjugated anti-human gammaglobulin (Cappel Laboratories); dilution medium: PBS (pH 7.2) + 0.05% Tween 20 + 2% bovine albumin; (Auer *et al*., 1981).
– Western blot (WB): Fasciola WB IgG®, LDBio Diagnostics, Lyon, France; (high-performance and specific confirmatory testing tool).
**2013–present**
– ELISA: DRG ELISA Fasciola IgG (human)^®^, DRG Instruments GmbH, Marburg, Germany; sensitivity 100%, specificity 100%.
– WB: Fasciola WB IgG^®^, LDBio Diagnostics, Lyon, France (high-performance and specific confirmatory testing tool).

DBP: di-butylphthalate; ELISA: enzyme-linked immunosorbent assay; FITC: fluorescein isothiocyanate; IgG: immunoglobulin G; PBS: phosphate-buffered saline.

### Statistical analysis

Descriptive statistics were applied to report on the individual cases included in the study. Variables were presented as numbers/frequencies and percentages.

## Results

### Baseline population characteristics and time of diagnosis

**Figure 1:**
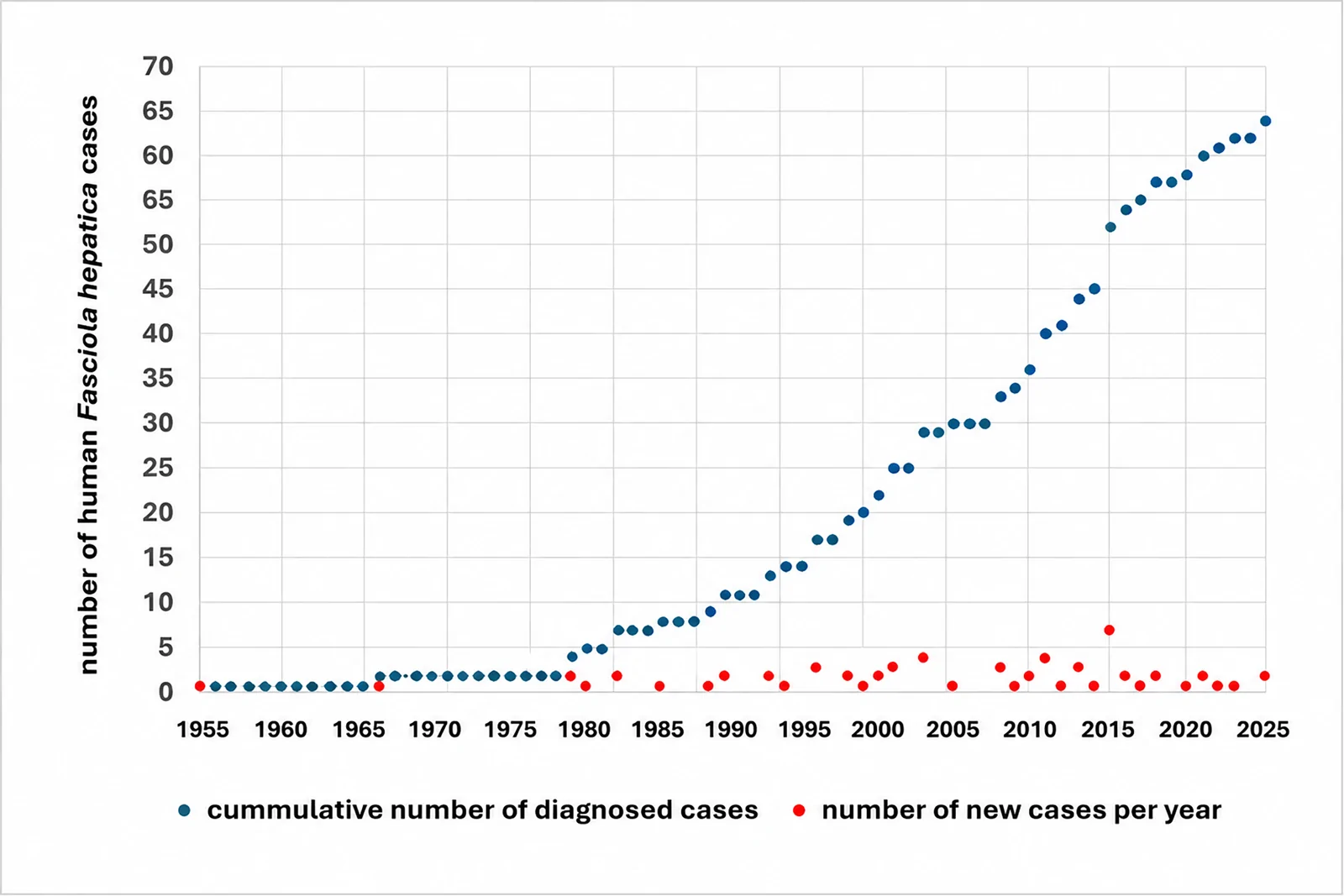
Diagnosed cases of *Fasciola hepatica* (FH) (red dots) in correlation to the year of diagnosis and the cumulative number of fasciolosis cases (blue dots) over time.

**Figure 2:**
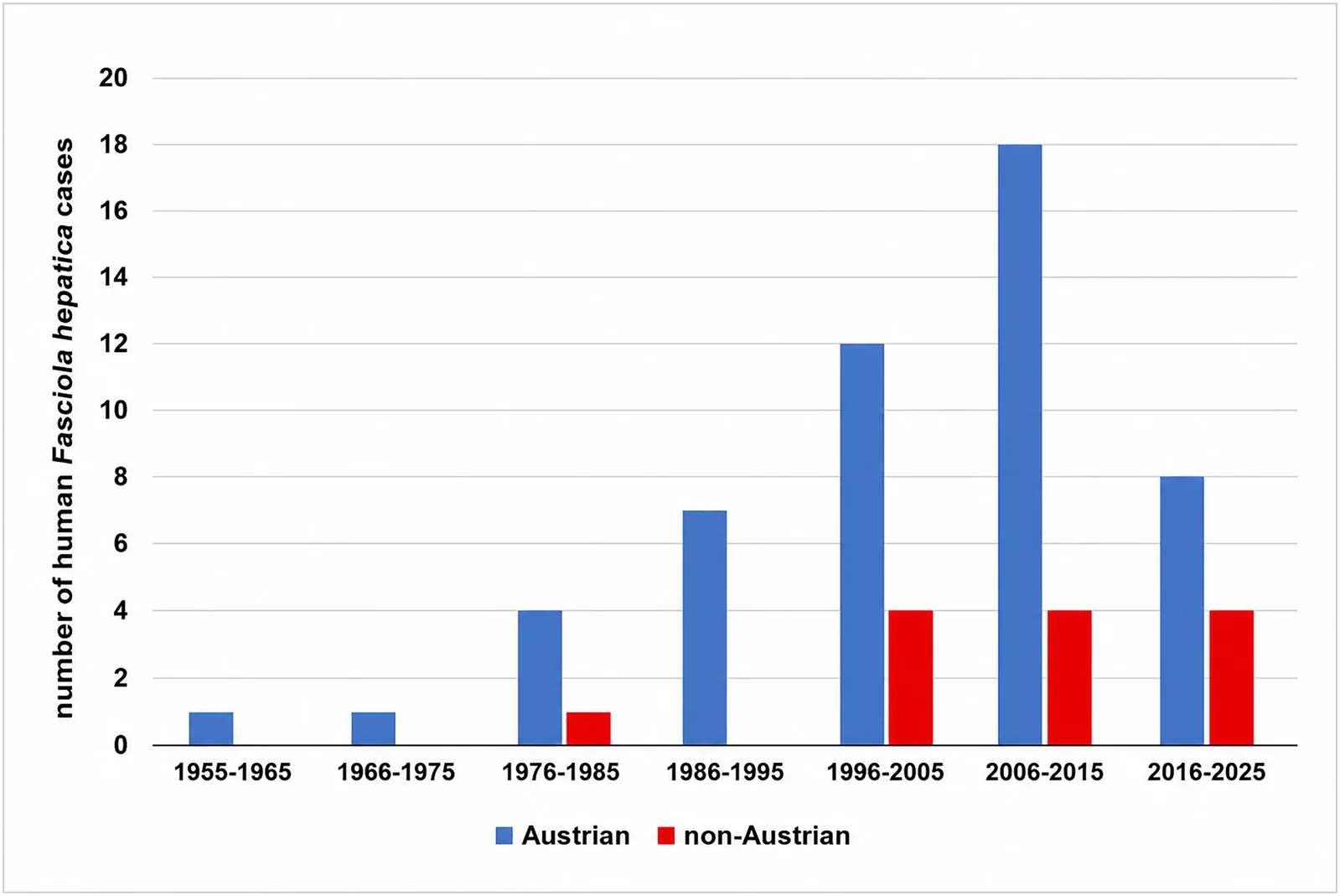
Number of human *Fasciola hepatica* infestations in Austria from 1955 to 2025 shown by 10-year intervals and split into individuals of Austrian and non-Austrian origin.

Between 1955 and 2025, 64 cases of human fascioliasis were diagnosed in Austria (Table 2, Fig. 1). This corresponds to an average of less than one case diagnosed per year. During the first years of the observational period, only a few human FH cases were diagnosed. Since 1980 the yearly diagnosed cases varied between 0 and 4, with one exceptional and unexplained peak of 7 cases in 2015 (Fig. 1). However, a clear decrease in diagnosed cases has been observed over the past 10 years (Fig. 2). Fifty-one (79.7%) of these 64 patients were of Austrian origin. Thirteen (20.3%) were of non-Austrian origin (Table 2): two were from countries of the former Soviet Union, three from Asia, four from Africa, and one from South America. The country of origin was not documented for four patients (Table 2). Gender distribution was nearly balanced, with 34 (53.1%) males and 30 (46.9%) females affected. The patients’ ages at diagnosis varied widely (range: 3–82 years, mean: 43.9 years) (Table 2).

**Table 2:** Human cases of *Fasciola hepatica* infestation diagnosed in Austria (1955–2025).

Patient No.^1^	Sex	Age at diagnosis	Year of diagnosis	Austrian federal state^2^	Origin	Possible risks of infection^3^	Travel history^4^	Symptoms and pathological laboratory tests^5^	Eosinophils^6^ relative %	Leucocyts^7^ cell/μL	Liver imaging^8^	Serology^9^	Eggs in stool^10^	Ref.
1	m	8	1955	UA	Austrian	NA	NSAB	AP/F/HA	>4%	21.000	NA	pos.	pos.	[7, 9]
2	m	NA	1967	ST	Austrian	NA	NSAB	NA	NA	NA	NA	NA	pos.	[7, 49]
3(a)	m	3	1980	UA	Austrian	FARM, cattle stool FH pos.	NSAB	AP/F/U/HSM	24%	27.000	NA	pos.	pos.	[5, 7]
4(b)	f	4	1980	UA	Austrian	FARM, cattle stool FH pos.	NSAB	AP/F/U/HSM	17%	15.000	NA	pos.	pos.	[5, 7]
5	f	28	1981	VI	Chilean	WATCR	Chile	AP/F/U/HA/NV/V	37%	< 10.000	NA	pos.	pos.	[3, 7, 38]
6	f	28	1983	ST	Austrian	NA	NSAB	AP/NV, ACS, LFT↑	> 4%	< 10.000	NA	NA	pos.	[7, 24]
7	m	75	1983	UA	Austrian	NA	NSAB	F/LFT↑, MEL, CHOL	56%	18.000	NA	pos.	pos.	[7]
8	m	46	1986	SA	Austrian	WATCR	NSAB	AP/F	19%	< 10.000	LL	pos.	pos.	[7, 75]
9	f	19	1989	BU	Austrian	NA	USA, CA BA	AP/SM	6%	> 10.000	NA	pos.	neg.	[7]
10(c)	m	77	1990	SA	Austrian	NA	NSAB	AP/F	> > 4%	> 10.000	NA	pos.	pos.	[7]
11(d)	f	65	1990	SA	Austrian	NA	NSAB	AP/F/LFT↑	> > 4%	> 10.000	LL	pos.	pos.	[7]
12	f	27	1993	SA	Austrian	NA	NA	ASYMP, LFT↑	18%	11.000	LL	pos.	neg.	[6]
13	m	55	1993	UA	Austrian	NA	GR	AP	39%	NA	LL	pos.	neg.	[6]
14	m	47	1994	SA	Austrian	NA	NSAB	F/LFT↑	55%	NA		pos.	neg.	[6]
15	m	59	1996	SA	Austrian	WATCR	Türkiye	F	20%	NA	LL	pos.	neg.	
16	m	69	1996	VI	Austrian	NA	SA, TUN, GR	F	NA	NA	LL	pos.	neg.	
17	f	40	1996	UA	Austrian	FARM, WATCR, cattle near	NSAB	AP	>4%	> 10,000	dil. DHC	pos.	neg.	[27]
						garden								
18	m	40	1998	SA	Austrian	veterinarian	NA	NA	NA	NA	NA	pos.	neg.	
19	m	9	1998	ANA	Non-Austrian	NA	NA	NA	NA	NA	NA	pos.	NA	
20	f	36	1999	VI	Austrian	NA	Türkiye	AP	66%	NA	NA	pos.	neg.	
21	m	41	2000	VI	Russian	NA	NA	NA	NA	NA	NA	pos.	neg.	
22	m	39	2000	VI	Iraqi	NA	NA	NA	NA	NA	NA	pos.	neg.	
23	m	38	2001	VO	Austrian	Forestry worker, WATCR	NSAB	WL/FAT/IgE↑	52%	15,000	LL	pos.	neg.	
24	m	78	2001	SA	Austrian	NA	NA	WL/INAPP	48%	14,000	LL	pos.	neg.	
25	f	45	2001	ANA	Austrian	NA	NA	NA	NA	NA	NA	pos.	neg.	
26 (e)	f	47	2003	VO	Austrian	NA	NA	AP/LFT↑	30%	NA	LL	pos.	neg.	
27(f)	f	80	2003	VO	Austrian	NA	NA	F	>4%	NA	LL	pos.	neg.	
28	m	11	2003	VI	Egyptian	NA	Egypt	ASYMP	31%	12,000	no LL	pos.	neg.	
29	f	60	2003	ST	Austrian	FARM, WATCR, ORV	NSAB	AP/WL	52%	> 10,000	LL	pos.	neg.	[37]
30	f	64	2005	VI	Austrian	WATCR	AS, PAC, LA	E	NA	12.000	NA	pos.	neg.	
31	m	31	2008	SA	Austrian	NA	NA	NA	NA	NA	NA	pos.	neg.	
32	f	50	2008	VO	Austrian	NA	NA	NA	NA	NA	NA	pos.	neg.	
33	m	62	2008	VO	Austrian	NA	SEUR, AF, AS, PAC	AP/WL/F/U	26%	NA	LL	pos.	neg.	
34	f	67	2009	UA	Austrian	FARM, cattle	NSAB	AP/F/NV/WL/IgE↑	19%	10,000	LL	pos.	neg.	
35	f	48	2010	TI	Austrian	wild garlic	NSAB	AP	68%	20,000	LL	pos.	neg.	
36	m	41	2010	VI	Turkish	WATCR, ORV	Türkiye	AP/FAT/IgE↑	56%	12,000	LL	pos.	neg.	[70]
37	m	35	2011	TI	Austrian	NA	Cyprus	ASYMP	40%	11,000	LL	pos.	neg.	
38	f	23	2011	VI	Ethiopian	NA	ETH, MOR	ASYMP	48%	NA	NA	pos.	neg.	
39(g)	m	64	2011	SA	Austrian	NA	Italy	AP/cholest. HP/P	6%	NA	LL	pos.	neg.	
40(h)	m	24	2011	SA	Austrian	NA	Italy	st.p. hep./SM/LF	8%	NA	LL	pos.	neg.	
41	m	23	2012	SA	Austrian	NA	NSAB	F	9%	NA	NA	pos.	neg.	
42	f	82	2013	TI	Austrian	NA	NSAB	AP/F/WL/C	18%	<10,000	LL	pos.	neg.	
43	f	26	2013	LA	Austrian	NA	Peru	AP/st. p. FH in Peru	40%	11,000	NA	pos.	neg.	
44	m	58	2013	TI	Austrian	NA	NSAB	AP/WL/C/LFT↑	50%	> 10,000	LL	pos.	neg.	
45	m	47	2014	CA	Austrian	FARM, WATCR	NSAB	AP/IgE↑	>4%	> 10,000	NA	pos.	neg.	
46	m	38	2015	TI	Somalian	NA	Somalia	AP/F/cough, SKL	8%	NA	NA	pos.	neg.	
47	f	41	2015	SA	Austrian	WATCR	Croatia	AP/F/LFT↑	> > 4%	18,000	LL	pos.	neg.	
48	f	71	2015	SA	Austrian	NA	NA	NA, LFT↑	> > 4%	NA	LL	pos.	neg.	
49	f	44	2015	TI	Georgian	NA	Georgia	AP/FH in Georgia	NA	NA	NA	pos.	neg.	
50	f	52	2015	UA	Austrian	NA	NA	NA	>50%	NA	LL	pos.	neg.	
51	f	79	2015	SA	Austrian	NA	NA	ASYMP	> > 4%	NA	NA	neg.	pos.	
52	m	10	2015	VI	Austrian	NA	NA	AP/D	> 4%	NA	NA	pos.	neg.	
53	f	67	2016	LA	Austrian	WATCR, garlic	Croatia, Cuba	AP/F/cough	57%	12.000	NA	pos.	neg.	
54	f	25	2016	ANA	Ethiopian	NA	NA	NA	24%	NA	NA	pos.	neg.	
55	f	54	2017	SA	Austrian	WATCR, cattle near garden	SEUR, AF, AS, PAC	ASYMP	> 4%	> 10.000	LL	pos.	neg.	
56	m	20	2018	UA	Austrian	vegan	Indonesia (Bali)	AP	30%	NA	LL	pos.	neg.	
57	m	55	2018	SA	Austrian	WATCR, sheep near garden	NSAB	C	> 4%	NA	NA	pos.	neg.	
58	f	76	2020	VI	non-Austrian	NA	NA	NA	NA	NA	NA	pos.	neg.	
59	m	36	2021	ANA	non-Austrian	NA	NA	NA	NA	NA	NA	pos.	NP	
60	m	47	2021	LA	Austrian	NA	ETH	NA	35%	NA	NA	pos.	neg.	
61	f	42	2022	SA	Austrian	NA	SEUR	NA	> > 4%	NA	NA	pos.	pos.	
62	m	29	2023	UA	Austrian	NA	NA	NA	23%	NA	NA	pos.	neg.	
63	m	20	2025	ANA	Afghan	NA	NA	AP	6%	NA	LL	pos.	NP	
64	f	42	2025	SA	Austrian	NA	NA	NA	NA	NA	NA	pos.	NP	

1) Relationship between patients: (a) brother of case 4, (b) sister of case 3, (c) husband of case 11, (d) wife of case 10, (e) relative of case 27, (f) relative of case 26, (g) father of case 40, (h) son of case 39. 2) Austrian federal states: VO: Vorarlberg; TI: Tyrol; SA: Salzburg; UA: Upper Austria; LA: Lower Austria; VI: Vienna; BU: Burgenland; ST: Styria; CA: Carinthia; ANA: allocation not available. 3) Possible risks of infection: WATCR: watercress; ORV: other raw vegetables; FARM: farmer. 4) Travel destinations: USA: United States of America; CA: Canada; BA: Bahamans; SEUR: southern Europe; GR: Greece; MOR: Morocco; TUN: Tunisia; SA: Saudi Arabia; ETH: Ethiopia; AF: Africa; AS: Asia; PAC: Pacific; LA: Latin America; NSAB: no stays abroad. 5) Symptoms and pathology laboratory: AP: abdominal pain F: fever; C: chills and flu-like symptoms; INAPP: inappetence; U: urticaria; E: erysipelas; SKL: skin lesions; HSM: hepatosplenomegaly; CHOL: cholangitis; P: pancreatitis; SM: splenomegaly; FAT: fatigue; WL: weight loss; D: diarrhea; HP: hepatopathy; LFT↑: elevated liver function tests; HA: headache; NV: nausea and vomiting; V: vertigo; LF: liver failure; ACS: acholic stool; MEL: melena; ASYMP: asymptomatic. 6) Eosinophils: normal range < 4%; >4%: (low) eosinophilia, but no exact data available; > > 4%: high eosinophilia, but no exact data available. 7) Leukocytosis: normal range 4,000–10,000/μL; <10,000: cell number within the normal range. 8) Liver imaging: LL: liver lesions; no LL: no liver lesions. 9) Serology tests for FH: Pos.: serological test positive for FH; neg.: serological test negative for FH; type of test depending on year applied, see Table 1 for details. 10) Stool tests for FH eggs: Pos.: eggs detected; neg.: eggs not detected; NP: not performed. NA: no data available.

### Geographic distribution of cases within Austria

**Figure 3:**
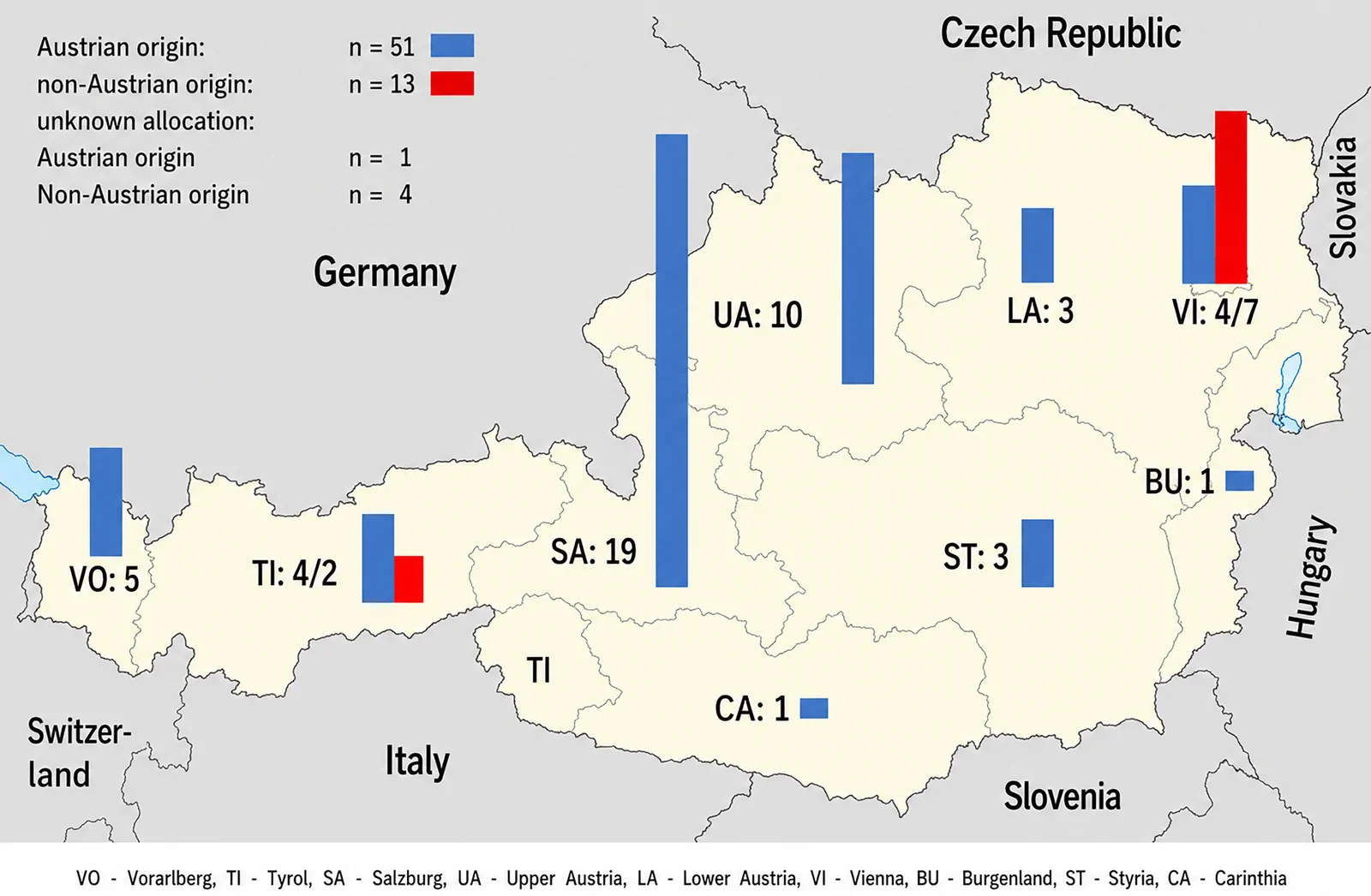
Geographic distribution of human *Fasciola hepatica* cases diagnosed between 1955 and 2025 by federal states of Austria. VO: Vorarlberg; TI: Tyrol; SA: Salzburg; UA: Upper Austria; LA: Lower Austria; VI: Vienna; BU: Burgenland; ST: Styria; CA: Carinthia.

Based on division into the nine Austrian federal states (Vienna, Lower Austria, Upper Austria, Salzburg, Tyrol, Vorarlberg, Styria, Carinthia, and Burgenland), cases were not distributed evenly (Fig. 3). The highest numbers of FH infestations were recorder in Salzburg (*n* = 19) and Upper Austria (*n* = 10), while Burgenland and Carinthia had each only one case (Fig. 3).

### Travel activities and migration

Travel activities were documented for 37 Austrian patients. Stays in FH-endemic regions outside of Austria were reported prior to FH diagnosis in 17/37 (45.9%) Austrian patients with documented travel activities (Table 2). Thus, it remains unclear whether FH infestation occurred in Austria or abroad. Until 1980, all affected individuals were Austrian. Since the 1990s, individuals with a non-Austrian background have also been diagnosed (Figs. 2 and 3). Most cases of non-Austrian origin were reported in Vienna (7/13; 53.85%), as immigrants predominantly settled in Vienna. Two cases of non-Austrian origin were documented in Tyrol (2/13; 15.38%), whereas geographic allocation of immigrants could not be evaluated in four cases (4/13; 30.77%) (Fig. 3).

### Behavioral risk factors

Watercress consumption was documented in 13/15 (86.67%) patients for whom nutritional history was available. One patient reported consuming wild garlic. Twenty-three patients (23/64; 35.93%) reported staying outside of Austria, either due to travel activities or due to migration from their home country to Austria (Table 2).

### Clinical presentation, documented clinical symptoms, and disorders

Clinical presentations and symptoms were documented for 48 patients (48/64; 75.0%). Six of them were asymptomatic (6/48; 12.5%), whereas the others reported a broad range of symptoms (Table 2). Fever (18/48; 37.5%) and weight loss, including inappetence (7/48; 14.58%), were the most common general symptoms, whereas chills and flu-like symptoms (3/48; 6.3%), fatigue (2/48; 4.2%), headaches (2/48; 4.2%), and vertigo (1/48; 2.1%) were less common. Various abdominal symptoms were reported, with abdominal pain (29/48; 60.4%) being the most frequent. Other symptoms and disorders included hepatosplenomegaly (2/48; 4.2%), hepatopathy (1/48; 2.1%), cholangitis (1/48; 2.1%), splenomegaly (2/48; 4.2%), pancreatitis (1/48; 2.1%), nausea and vomiting (2/48; 4.2%), diarrhea (1/48; 2.1%), acholic stool (1/48; 2.1%), and melena (1/48; 2.1%). Skin symptoms, mainly urticaria (6/48; 12.5%), and pulmonary symptoms, including cough (2/48; 4.2%), were other infrequently documented medical conditions (Table 2).

### Laboratory data

Leucocyte counts were reported in 28 cases (28/64; 43.75%) and eosinophils counts in 50 cases (50/64; 78.13%). All available results for leucocytes (leucocytes >10,000/μL) and for the relative eosinophil percentage were abnormally increased. The relative eosinophil percentage was significantly increased in many cases (maximum: 68 rel.%). Elevated liver parameters were reported (*n* = 9) as well as increased immunoglobulin E (*n* = 3) (Table 2).

### Liver imaging data

Liver imaging data (sonography and/or computed tomography) were available in 28/64 patients (43.75%). Of these patients, 26 had abnormal liver lesions (92.8%), one showed no liver lesions, but a dilated common bile duct, and only one had normal liver imaging results (3.6%) (Table 2).

### Serological and coprological testing confirming FH infestation

FH diagnosis was either performed and confirmed by different serological tests (Table 1) and/or stool tests in all patients. To be included in the study as a patient with FH, at least one of the methods had to yield a positive result. Serological FH tests were performed in 62 patients (62/64; 96.88%), with 61 positive cases (61/62; 98.39%).

Stool samples were collected from 60 patients (60/64; 93.75%), but were positive in only 12 cases (12/60; 20.0%). Most positive stool samples were collected during the first years of the period (Table 2). In recent years, stool samples were no longer collected from all patients.

## Discussion

Fascioliasis is a parasitosis frequently found in animals worldwide [48]. Humans are mostly affected in endemic areas, with 35 to 72 million people being infected, and an estimated 180 million people at risk of infection, according to the WHO [20, 63, 74]. However, in high income countries, such as Austria, fascioliasis rarely affects humans and can be considered an exotic disorder. During the 70-year observation period covered by this retrospective analysis, the first case report of human FH infestation in Austria was published by Bergsmann *et al.* in 1957 [9]. A preliminary overview of FH in Austria was published in 1968 [67], followed by other articles [3, 5–7, 24, 27, 37, 38, 49, 70]. The most recent publication, in 2014, presented an overview of trematode-caused diseases in Central Europe and included FH epidemiology in Austria [4].

Our study revealed 64 human FH infestation cases in Austria between 1955 and 2025. Although the overall diagnostic rates were low, making human FH a rare disease in Austria, they increased steadily over the decades for several reasons (Fig. 2). First, diagnostic tests and availability changed and improved over the years [4, 8] (Table 1). Until 1980, in routine clinical practice, diagnosis of human FH infestation was mainly based on FH egg detection in stool samples. Thus, the low sensitivity of this diagnostic method may explain the low number of only two reported FH cases during the early years of the period (Fig. 1). Improved diagnostic approaches over time, through the introduction of highly sensitive and specific serological testing, is reflected by the clear increase in detected cases since 1980 (Figs. 1 and 2). Second, the medical health care system has improved constantly over the years in Austria. This improvement has been based on increased availability of medical care, including routine medical checks, access to diagnostic facilities, and most likely increased knowledge about disorders rarely seen in Austria, such as human FH. This may explain that even some asymptomatic patients (Table 2; cases 12, 28, 37, 38, 51, 55) were diagnosed with FH. Eosinophilia was common to all these cases, in one case up to 48%, which led the clinician to the diagnostic work-up of a possible parasitic disorder.

However, the reason for the clear decrease of diagnosed cases during the last 10 years as compared to previous years remains unclear. One reason may be demographic changes. In recent decades, the percentage of people living in rural areas has changed. For example, an increase in human infestation was observed in the Limousin region of central France between 1955 and 1987, with a subsequent decrease until 1998 [61]. The authors attributed this to demographic changes in the region: 66.3% of people lived in villages before 1980 compared to 19.2% after 1981. A similar effect may be responsible for the decrease in FH cases in recent years in Austria.

Watercress was identified as the infection source in 98% of human FH infestations in the Limousin region of Central France [61]. Tartar *et al.* [68] also found a history of watercress consumption in 63.2% of 15 human FH infestation cases in Türkiye. Although not comprehensively documented in our study population, eating habits were available for 15 cases, and a history of consumption of watercress, wild garlic, or other raw vegetables was documented in 14 cases. This finding is thus in line with the known fact that eating raw vegetables, particularly watercress, frequently represents the mode of FH transmission to humans. This highlights the need to document eating habits as a potentially important risk behavior in medical histories. Nonetheless, changes in eating habits and the source of acquisition of vegetables in recent years may represent a further cause of lower numbers of diagnosed FH cases. Nowadays, it is possible that people tend to eat fewer self-grown vegetables, which are potentially contaminated by metacercariae, and instead consume vegetables bought in food markets and not contaminated by metacercariae.

Finally, another factor leading to changes in the number of human FH infestations in Austria over time may be climate change, with altered rainfall distributions and rising temperatures being major predictors [34]. These predictors are used to develop temporal forecasting systems for FH infestations among dairy animals in different countries [22, 47]. Changes in rainfall intensity, with a reduction in wet biotopes harboring watercress species, giving FH fewer biotopes for reproduction and transmission to humans, may explain the lower infestation numbers in recent years. However, climate effects should be evaluated differently for Austria because climate shows broad regional variation, as Austria is a mainly mountainous country. The higher incidence of FH infestations observed in some Austrian regions may therefore be associated with regional differences in climate. The federal states predominantly affected by FH (Vorarlberg, Tyrol, Salzburg, and Upper Austria) are located in western and northern Austria, with more frequent and higher rainfall amounts compared to southern and eastern Austria. Moreover, the rising number of FH diagnoses until 2015 may be due to improvements in the healthcare system, particularly regarding FH diagnostic tests. However, these hypotheses require further study.

Since the 1980s, individuals of non-Austrian origin have accounted for increasing numbers of human FH cases, reflecting increasing immigration to Austria. The migration of people from FH-endemic areas to regions with low FH incidence has only been addressed by a few studies [25, 30, 53, 69, 72]. Jensenius *et al.* [30] reported three cases of immigrants with human FH infestation (Vietnam: *n* = 2 and Ethiopia: *n* = 1) in Norway, Velev *et al.* [72] reported the case of a Syrian child in Bulgaria, and Noyer *et al.* [53] and Graham *et al.* [25] reported human FH infestation in one and two immigrants in the United States, respectively. A large observational [69] retrospective study found 16 FH cases imported to Spain between 2009 and 2019 (six immigrants, four people visiting friends and relatives abroad, and two travelers). Picher and Aspöck [55] found a high helminthic index of 63% in 100 Vietnamese refugees when testing for several helminths and protozoa. However, they found no FH infestation. Thus, in countries with low incidence of human FH infestation, the disease may often be overlooked [53] and imported fascioliasis is rarely reported in these countries [25, 30].

Aside from migration, the travel habits of the local population must be considered when discussing FH. Importantly, overall travel has increased significantly in developed countries worldwide over our observation period. This can expose people from developed countries to FH when traveling to FH-endemic areas. Consequently, human fascioliasis can be considered an important parasitic disease in travel medicine [2, 33]. Therefore, traveling to FH-endemic regions may also have contributed to human FH infestations in Austrian residents.

So far, only a few cases of human FH infestation in patients who traveled to FH-endemic areas have been reported [31, 33, 47, 50, 64]. Possible FH exposure during travel should be considered by clinicians in countries where human fascioliasis is rare, when examining patients presenting with symptoms such as unclear abdominal pain, fever, or eosinophilia, who have a corresponding travel history. Increased awareness of FH infestations is needed [54] and a possible parasitic infestation should be suspected in patients with travel history to endemic areas [30]. Obtaining a detailed travel history is therefore necessary and essential to achieve a timely FH diagnosis [25].

Stays in FH-endemic regions outside of Austria were documented prior to FH diagnosis in 45.9% of patients with documented travel, but due to the retrospective study design, it remains unclear whether these patients were infested in Austria or abroad. Furthermore, as some of the patients traveled to subtropical or tropical regions of Africa and Asia, also endemic for *F. gigantica*, an infestation by this fluke species cannot be ruled out in some patients documented with fascioliasis (the serological tests applied are highly sensitive for fascioliasis, but cannot discriminate between FH and *F. gigantica*). For these cases with a travel history to tropical and subtropical Africa and Asia, the additional application of various molecular methods for *Fasciola* DNA testing may also be considered in countries with a low fascioliasis incidence, such as Austria, in order to further improve and diversify FH diagnostics.

Our study has some limitations. The retrospective design provided incomplete datasets for several patients diagnosed with FH, thus limiting data interpretation regarding certain topics. Moreover, with a 70-year observational period, the healthcare system changed dramatically, not only in Austria, but worldwide. Diagnostic tools, such as testing and imaging methods for diagnosing FH, either changed during the observational period or were not available in early years. As a result, underreporting of FH cases during the early years cannot be ruled out. However, the 70-year observational period covering the whole country provided an overall view on human FH infestations in Austria. Despite the abovementioned limitations, demographic hot spots within Austria, at-risk groups (immigrants and travelers coming from or traveling to endemic FH areas), and behavioral factors (eating watercress or raw vegetables) were outlined.

In conclusion, human FH infestation remains a rare parasitic disease in Austria, even after the diagnostic improvements achieved over the years and the introduction of highly sensitive serological testing. Moreover, immigration from and increased travel to regions with endemic fascioliasis likely contributed to the cases detected in Austria during our observation period. Some Austrian federal states seem to be more affected by human FH infestation than others, possibly due to climatic reasons. Despite the low incidence of human FH infestation in countries such as Austria, clinicians should be aware of FH infestation as a rare disease and consider it a differential diagnosis in patients with unclear abdominal symptoms and eosinophilia, especially if the patient has a travel history, to facilitate a timely diagnosis and avoid potentially life-threatening complications.

## Data Availability

Data are available upon reasonable request to the corresponding author.
